# Associations between gestational weight gain and preterm birth in Puerto Rico

**DOI:** 10.1186/s12884-020-03292-1

**Published:** 2020-10-07

**Authors:** Stephanie M. Eick, Michael Welton, Mechelle D. Claridy, Skarlet G. Velasquez, Nicholas Mallis, José F. Cordero

**Affiliations:** Department of Epidemiology and Biostatistics, College of Public Health, University of Georgia, 101 Buck Road, GA Athens, United States

**Keywords:** Preterm birth, Health disparities, Puerto Rico, Pregnancy, Weight gain, Body mass index

## Abstract

**Background:**

Preterm birth (PTB; gestational age < 37 weeks) is the leading cause of infant morbidity and mortality worldwide. Low and excessive gestational weight gain (GWG) have been previously cited as risk factors for PTB, however the magnitude of association varies across populations. No studies have examined low and excessive GWG as modifiable risk factors for PTB in Puerto Rico, an area with inexplicably high PTB rates.

**Methods:**

To examine the relationship between GWG and PTB, we conducted a retrospective analysis using birth certificate data files from the Puerto Rico Department of Health from 2005 to 2012. GWG was standardized to a 40-week gestational duration and was categorized into low, adequate, or excessive for each category of pre-pregnancy body mass index using American College of Obstetricians and Gynecologists guidelines. Logistic regression was used to determine the crude and adjusted odds ratios (OR) and 95% confidence intervals (CI) for the association between GWG and PTB.

**Results:**

There were 320,695 births included in this analysis; 40.6% with high GWG and 27.3% with low GWG. A greater percentage of women with low GWG were less than 20 years of age, had less than a high school education, and were underweight compared to women with adequate and excessive GWG. Women with low compared to adequate GWG had increased odds of PTB (OR = 1.34, 95% CI = 1.30–1.37). However, excessive compared to adequate GWG was not associated with PTB (OR = 0.99, 95% CI = 0.97–1.02).

**Conclusions:**

Among women in Puerto Rico, low GWG was associated with increased odds of PTB. With the exception of obesity, these associations persisted within all strata of pre-pregnancy body mass index, highlighting the importance of maintaining a healthy weight during pregnancy. Future research should examine other factors that may contribute to GWG, such as dietary nutrients, and explore pathways through which GWG may be contributing to PTB.

## Background

Preterm birth (PTB), defined as gestational age less than 37 weeks, is one of the leading causes of infant morbidity and mortality worldwide [1]. In addition to a high societal cost, babies born preterm are at risk for numerous health problems both at birth and later in life [1]. Rates of PTB in Puerto Rico have been some of the highest in the U.S. and peaked at one of the highest rates in the world. Puerto Rico’s PTB rate reached 19.9% in 2006 and subsequently declined to 11.4% in 2017 [2, 3]. Nonetheless, Puerto Rico received a ‘D’ rating by the March of Dimes for its 2017 PTB rate, which remains high compared to the continental U.S. [3].

Gestational weight gain (GWG) may be one factor contributing to Puerto Rico’s high PTB rate. A recent systematic review showed that women who gained less than the recommended weight during pregnancy had increased odds of PTB [4]. The magnitude of the association was strongest for underweight women, but associations persisted within all strata of body mass index (BMI) [4]. Furthermore, the associations between low weight gain during pregnancy are greater in magnitude for very PTB compared to moderately PTB and the strength of association decreases as BMI increases [5]. It is hypothesized that low GWG contributes to PTB through deficiencies in micro and macronutrients, which increase the risk of PTB [6]. Inadequate GWG has also been associated with increased odds of infant death [7] and small for gestational age births [4].

The associations between excessive GWG and PTB are less understood. Overweight and obesity are some of the strongest predictors of excessive GWG [8] and an estimated 40% of pregnant women in Puerto Rico were overweight or obese between 2005 and 2012 [9]. Excessive GWG has been associated with PTB, however associations remain inconsistent and population-specific [4, 10]. For example, excessive GWG among women in China was associated with a 27% increase in risk of PTB [10]. In comparison, weight gain above the target guidelines was associated with a 17% reduction in risk of PTB among women in the U.S. and Europe [11]. Excessive GWG may be especially important in Puerto Rico, as previous research has shown that 32% of obese pregnant women in Puerto Rico have excessive GWG [12], this is a larger percentage of obese women with excessive GWG compared to other populations [13, 14]. Excessive GWG has also been implicated as playing a role in the short and long term health of infants. For example, infants born to mothers who gain above the recommended weight during pregnancy are more likely to be born large for gestational age, have macrosomia, and go on to develop childhood obesity [15, 16].

While there have been studies that examined the associations between GWG and PTB, these associations have not been explored in Puerto Rico. Previous literature has shown that the association between GWG and PTB varies across cultures and ethnicities [11] and the results have not been consistent. Previously, we identified an association between overweight, obesity and PTB in Puerto Rico using birth certificate data [9]. In the present study, we built upon this work and examined associations between GWG and PTB and examined how these relationships varied based on pre-pregnancy body mass index (PP-BMI). We hypothesized that women with inadequate and excessive GWG would have increased odds of PTB.

## Methods

### Study population

The data source for this analysis was the Puerto Rico birth certificate data files which were obtained from the Vital Statistics Office of the Puerto Rico Department of Health. Birth certificates contain a variety of demographic and clinical information from all births occurring in Puerto Rico from 2005 to 2012. For this analysis, we restricted to singleton births since multiple gestations is a known risk factor for preterm delivery. We additionally restricted our analysis to women who were residents of Puerto Rico at the time of delivery to ensure generalizability to women on the island.

This analysis was reviewed by the University of Georgia’s Institutional Review Board (IRB ID: STUDY00006591) and was determined to be exempt because we use secondary data that has been deidentified.

### Preterm birth

Gestational age information was provided on the birth certificate. We categorized gestational age into preterm (< 37 weeks gestation) and full term (≥ 37 weeks gestation) birth. We further categorized PTB into: late preterm (34 weeks to 36 and 6 days), moderately preterm (32 weeks to 33 weeks and 6 days), and very preterm (< 32 weeks) [17]. We excluded infants with implausible or extreme gestational age (< 23 weeks gestation and > 41 weeks gestation) in our analyses. Infants born prior to 23 weeks gestation were excluded as deliveries at this gestational age typically result in neonatal death [18].

### Pre-pregnancy body mass index

PP-BMI was calculated based on maternal pre-pregnancy height and weight. PP-BMI was categorized into underweight (< 18.5 kg/m^2^), normal (18.5–24.9 kg/m^2^), overweight (25-29.9 kg/m^2^), and obese (≥ 30 kg/m^2^) based on standard World Health Organization (WHO) classifications [19].

### Gestational weight gain

GWG was calculated by subtracting pre-pregnancy weight from maternal weight at delivery. Our analysis used an adjusted GWG measure which was standardized to a 40-week gestational duration. This established method has been used previously where GWG was divided by gestational age at delivery and then multiplied by 40 [20]. The association between GWG and PTB may be confounded by gestational age, as women who deliver preterm may have had less time to gain weight during pregnancy. Therefore, we utilized a standardized measure of GWG in our analysis which helps alleviate this confounding. This adjustment is necessary in order to account for the fact that women are able to gain more weight the longer they are pregnant [21].

We then categorized the adjusted GWG estimate into either low, adequate, or excessive for each category of PP-BMI based on American College of Obstetricians and Gynecologists (ACOG) classifications [22]. ACOG recommendations are based on Institute of Medicine (IOM) guidelines. In 2009, the IOM published revised GWG guidelines that are based on PP-BMI by the WHO and are independent of age, parity, smoking history, race, and ethnic background [16]. The GWG guidelines attempt to balance the risks of having large for gestational age infants, small for gestational age infants, preterm births, and postpartum weight retention. For underweight women, those who gained between 28 and 40 lbs were considered to have adequate weight gain. Normal weight women who gained between 25 and 35 lbs had adequate weight gain. Overweight and obese women who gained between 15 and 25 lbs and 11 and 20 lbs, respectively, had adequate weight gain. Women were considered to have low weight gain if they gained fewer pounds than the target range and were considered to have excessive weight gain if weight gain was above the target range for each category of PP-BMI.

### Statistical analyses

Frequencies and counts were used to describe our study population. We included the following covariates in our analyses: maternal age in years (< 20, 20–24, 25–29, 30–35, ≥ 35), PP-BMI (underweight, normal, overweight, obese), marital status (single, married or living with a partner), maternal education (< high school, high school or equivalent, >high school), insurance status (public, private, other/out of pocket/charity), and birth weight for gestational age. Birth weight for gestational age was categorized as small for gestational age (SGA; birth weight < 10th percentile for gestational age), average for gestational age (AGA; birth weight between 10th and 90th percentiles for gestational age), and large for gestational age (LGA; >90th percentile for gestational age) using a U.S. based population reference [23]. Other studies conducted in Puerto Rico have used these same percentiles to calculate birthweight for gestational age [24]. We examined the distribution of covariates within GWG categories (low, adequate, and excessive).

Logistic regression was used to calculate crude and adjusted odds ratios (ORs) and 95% confidence intervals (CI) for the associations between demographic characteristics and GWG and for the associations between GWG and PTB. Covariates retained in adjusted logistic regression models were associated with both GWG and PTB in bivariate analyses and had evidence in the literature supporting an association between exposure and outcome. Final models were adjusted for maternal age, maternal education, marital status, and birth weight for gestational age. We assessed for collinearity between all covariates included in adjusted models by examining condition indices and variance decomposition proportions and found no evidence of collinearity between variables included in adjusted models.

In addition, we examined associations between GWG and PTB within strata of PP-BMI in separate logistic regression models. As a sensitivity analysis, we also examined associations between GWG and PTB by PTB subtypes as previous research has shown that the combined effect of GWG and PP-BMI is greater for very preterm compared to moderately preterm infants [5]. Adequate GWG was the reference group for all models.

P-values < 0.05 were considered statistically significant and all statistical analyses were done using SAS 9.4 (Cary, NC).

## Results

**Fig. 1:**
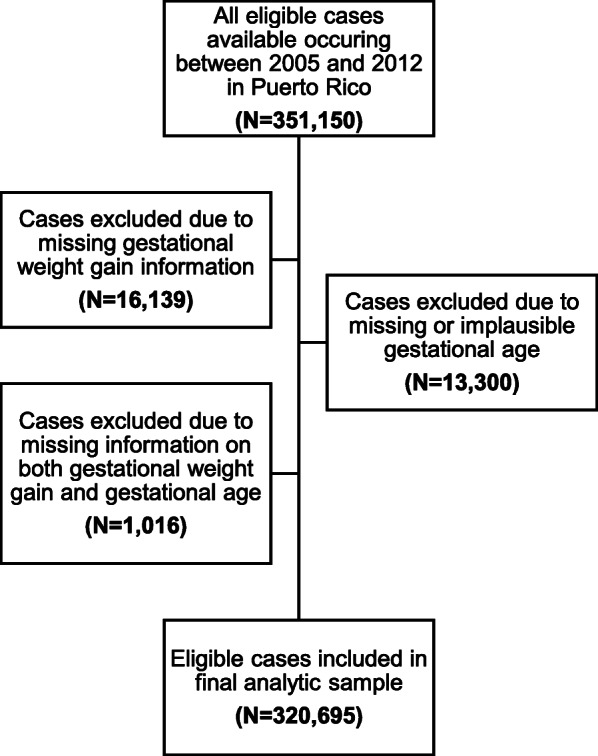
Flow chart indicating the inclusion of selected births into final analytic sample

There were 351,150 singleton births among women living in Puerto Rico between 2005 and 2012. We excluded 16,139 women due to missing GWG information. Of this group, 767 of these observations were lost due to inconsistencies in maternal height or weight, 13,300 observations were lost due to missing or implausible gestational age, and 1,016 were lost due to missing information on both GWG and gestational age. This left 320,695 women who were included in the current analysis (Fig. 1). Within our analytic sample, 56,258 (17.5%) delivered preterm. There were 43,360 (13.5%), 6,123 (1.9%), and 6,775 (2.1%) infants who were born late, moderately, and very preterm, respectively. The majority of women in this analysis had excessive GWG (40.6%) and a slightly smaller percentage of women had adequate GWG (32.1%) and low GWG (27.3%). Similar to other reported rates of PTB in Puerto Rico, our study population had a PTB rate of 18.9% in 2005 which decreased to 16.3% in 2012.

The largest percentages of women in our analytic sample were between 20 and 24 years of age (32.5%), were normal weight (49.7%), and had greater than a high school education (48.5%). Significant differences between demographic characteristics were observed when comparing women with low and excessive GWG to women with adequate GWG (Table 1). For example, women with low GWG were more likely to be single (34.6% vs. 39.4%) and have less than a high school education (22.8% vs. 18.0%). Similarly, a lower number of women with excessive GWG had public insurance (66.3% vs. 73.4%) and a greater number of women with excessive GWG delivered a LGA infant (10.0% vs. 5.78%) (Table 1).

**Table 1:** Distribution of gestational weight gain across demographic characteristics and pregnancy outcomes, Puerto Rico 2005–2012 (N = 320,695)

	Low (*N* = 87,580)		Adequate (*N* = 103,085)	Excessive (*N* = 130,030)	
	N (%)	OR (95% CI)	N (%)	N (%)	OR (95% CI)
Maternal Age (years)					
< 20	18,322 (20.9)	1.11 (1.08, 1.14)	18,588 (18.0)	21,033 (16.2)	0.92 (0.90, 0.95)
20–24	29,681 (33.9)	Ref	33,409 (32.4)	40,999 (31.5)	Ref
25–29	20,525 (23.4)	0.89 (0.87, 0.91)	25,916 (25.1)	34,984 (26.9)	1.10 (1.08, 1.12)
30–34	13,944 (15.9)	0.84 (0.82, 0.87)	18,594 (18.0)	24,913 (19.2)	1.09 (1.07, 1.12)
≥35	5,093 (5.82)	0.87 (0.84, 0.91)	6,570 (6.37)	8,090 (6.22)	1.00 (0.97, 1.04)
Missing	15 (0.02)		8 (0.01)	11 (0.01)	
Pre-pregnancy BMI					
Underweight (< 18.5 kg/m^2^)	10,768 (12.3)	1.13 (1.10,1.16)	9,923 (9.63)	4,440 (3.41)	0.50 (0.49, 0.52)
Normal (18.5–24.9 kg/m^2^)	53,777 (61.4)	Ref	55,922 (54.3)	49,685 (38.2)	Ref
Overweight (25-29.9 kg/m^2^)	12,380 (14.1)	0.58 (0.56, 0.59)	22,322 (21.7)	44,650 (34.3)	2.25 (2.21, 2.30)
Obese (≥ 30 kg/m^2^)	10,655 (12.2)	0.74 (0.72, 0.76)	14,918 (14.5)	31,255 (24.0)	2.36 (2.30, 2.41)
Marital Status					
Single	30,269 (34.6)	0.81 (0.80, 0.83)	40,588 (39.4)	51,220 (39.4)	1.00 (0.98, 1.02)
Married/Living Together	57,300 (65.4)	Ref	62,485 (60.6)	78,799 (60.6)	Ref
Missing	11 (0.01)		12 (0.01)	11 (0.01)	
Education					
< High School	19,943 (22.8)	1.19 (1.16,1.22)	18,590 (18.0)	21,806 (16.8)	0.93 (0.91, 0.95)
High School	29,803 (34.0)	Ref	33,056 (32.1)	41,758 (32.1)	Ref
>High School	37,675 (43.0)	0.81 (0.80,0.83)	51,285 (49.8)	66,286 (51.0)	1.02 (1.00, 1.04)
Missing	159 (0.18)		154 (0.15)	180 (0.14)	
Insurance Status^a^					
Public	64,239 (73.4)	1.33 (1.31, 1.36)	69,644 (67.6)	86,247 (66.3)	0.94 (0.93, 0.96)
Private	22,184 (25.3)	Ref	32,118 (31.2)	42,160 (32.4)	Ref
Other	921 (1.05)	1.30 (1.18, 1.42)	1,029 (1.00)	1,298 (1.00)	0.96 (0.88, 1.04)
Missing	236 (0.27)		294 (0.29)	325 (0.25)	
Preterm Birth					
Yes	16,722 (19.1)	1.25 (1.22, 1.28)	16,329 (15.8)	23,207 (17.9)	1.15 (1.13, 1.18)
No	70,858 (80.9)	Ref	86,756 (84.2)	106,823 (82.2)	Ref
Birth Weight for Gestational Age					
Small for Gestational Age	11,503 (13.1)	1.45 (1.41, 1.49)	9,649 (9.36)	8,674 (6.67)	0.73 (0.70, 0.75)
Adequate for Gestational Age	71,905 (82.1)	Ref	87,459 (84.8)	108,289 (83.3)	Ref
Large for Gestational Age	4,143 (4.73)	0.85 (0.81, 0.88)	5,958 (5.78)	13,034 (10.0)	1.77 (1.71, 1.82)
Missing	29 (0.03)		19 (0.02)	33 (0.03)	

Note: Adequate is the reference group for odds ratios

*Abbreviations*: *OR O*dds ratio, *CI *Confidence interval, *Ref *Reference, *BMI *Body mass index

^a^Other includes out of pocket, charity, and other

In adjusted models not restricted to strata of PP-BMI, women with low compared to adequate GWG had increased odds of PTB (OR = 1.34, 95% CI = 1.30, 1.37) (Table 2). Low GWG was additionally associated with increased odds of PTB across nearly all strata of PP-BMI, although OR estimates were lower in magnutide as PP-BMI increased (Table 2). For example, underweight women with low GWG had a 1.56 increase in odds of PTB (95% CI = 1.45, 1.68) compared to underweight women with adequate GWG. The corresponding OR for overweight women with low compared to adequate GWG was 1.29 (95% CI = 1.21, 1.37).

**Table 2:** Odds ratios and 95% confidence intervervals for the associations between gestational weight gain and preterm birth overall and within strata of body mass index

		Body Mass Index			
	**Total**	**Underweight**	**Normal**	**Overweight**	**Obese**
Gestational Weight Gain	**OR (95% CI)**	**OR (95% CI)**	**OR (95% CI)**	**OR (95% CI)**	**OR (95% CI)**
**Adjusted**					
Low	1.34 (1.30, 1.37)	1.56 (1.45, 1.68)	1.39 (1.35, 1.44)	1.29 (1.21, 1.37)	1.00 (0.93, 1.07)
Adequate	Ref	Ref	Ref	Ref	Ref
Excessive	0.99 (0.97, 1.02)	0.99 (0.89, 1.09)	1.06 (1.02, 1.10)	0.98 (0.94, 1.03)	0.94 (0.89, 0.99)
**Unadjusted**					
Low	1.25 (1.22, 1.28)	1.33 (1.25, 1.43)	1.32 (1.27, 1.36)	1.25 (1.18, 1.32)	0.95 (0.89, 1.01)
Adequate	Ref	Ref	Ref	Ref	Ref
Excessive	1.15 (1.13, 1.18)	1.19 (1.09, 1.30)	1.25 (1.21, 1.29)	1.10 (1.06, 1.15)	1.03 (0.98, 1.08)

Note: models adjusted for maternal age, maternal education, marital status, and birth weight for gestational age

*Abbreviations*: *OR *Odds ratio, *CI *Confidence interval, *Ref *Reference

In crude analyses, excessive compared to adquate GWG was significantly associated with increased odds of PTB (OR = 1.15, 95% CI = 1.13, 1.18) (Table 2). However, the associations between excessive GWG and PTB were generally attenauated to non-significance in adjusted models (Table 2).

Associations between GWG, PP-BMI, and PTB subtypes are presented in Table 3. Within all strata of PP-BMI and across PTB subtypes, low GWG was associated with increased odds of PTB. The only exception was for late preterm births, where low GWG was not associated with preterm birth among obese women (OR = 0.93, 95% CI = 0.87, 1.01). The association that was the greatest in magnitude was for underweight women with low GWG, who had a 2.08 (95% CI = 1.66, 2.61) increase in odds of very PTB compared to underweight women with adequate GWG. Obese women with low GWG were also more likely to deliver very preterm compared to obese women with adequate GWG, although point estimates were not as large (OR = 1.44, 95% CI = 1.21, 1.72). Excessive compared to adequate GWG was not associated with odds of very, moderately, late PTB (Table 3).

**Table 3:** Adjusted odds ratios and 95% confidence intervervals for the associations between gestational weight gain and preterm birth categories overall and within strata of body mass index

		**Body Mass Index**			
	**Total**	**Underweight**	**Normal**	**Overweight**	**Obese**
Gestational Weight Gain	**OR (95% CI)**	**OR (95% CI)**	**OR (95% CI)**	**OR (95% CI)**	**OR (95% CI)**
**Very Preterm**					
Low	1.84 (1.71, 1.97)	2.08 (1.66, 2.61)	2.07 (1.88, 2.29)	1.73 (1.47, 2.04)	1.44 (1.21, 1.72)
Adequate	Ref	Ref	Ref	Ref	Ref
Excessive	0.94 (0.88, 1.00)	0.91 (0.69, 1.21)	1.05 (0.95, 1.16)	0.90 (0.79, 1.04)	0.95 (0.83, 1.10)
**Moderately Preterm**					
Low	1.50 (1.40, 1.61)	1.84 (1.48, 2.29)	1.61 (1.46, 1.77)	1.31 (1.11, 1.56)	1.24 (1.03, 1.51)
Adequate	Ref	Ref	Ref	Ref	Ref
Excessive	0.83 (0.78, 0.89)	0.82 (0.62, 1.09)	0.93 (0.84, 1.02)	0.77 (0.67, 0.88)	0.96 (0.82, 1.11)
**Late Preterm**					
Low	1.28 (1.24, 1.31)	1.47 (1.36, 1.59)	1.33 (1.28, 1.38)	1.25 (1.17, 1.33)	0.93 (0.87, 1.01)
Adequate	Ref	Ref	Ref	Ref	Ref
Excessive	1.00 (0.97, 1.02)	0.99 (0.89, 1.10)	1.06 (1.02, 1.10)	1.00 (0.95, 1.05)	0.93 (0.88, 0.98)

Note: models adjusted for maternal age, maternal education, marital status, and birth weight for gestational age

*Abbreviations*: *OR *Odds ratio, *CI *Confidence interval, *Ref *Reference

## Discussion

Our analysis identified low GWG as an important, modifiable risk factor for PTB in Puerto Rico. These associations became more pronounced after controlling for confounders and persisted within all strata of PP-BMI, providing strong evidence that the relationship between GWG and PTB is, in part, independent of PP-BMI. Results from our study contribute to the growing body of literature identifying pre-pregnancy weight and GWG as modifiable risk factors for adverse pregnancy outcomes.

A unique aspect of our study was that we examined associations between GWG and PTB within categories of PP-BMI. In our study, low GWG was associated with increased odds of PTB across all strata of PP-BMI. Our study also showed that the odds of PTB among pregnant women with low GWG were highest in underweight women, followed by women with normal, overweight, and obese PP-BMI. This finding is consistent with a recent systematic review of women in the U.S. and Europe showing that normal weight, overweight, and obese women with GWG below the target guidelines have increased odds of delivering preterm [11]. The positive association between low GWG and PTB was suggestive among underweight women in that review, but was not statistically significant. Nonetheless, our finding that underweight women with low GWG have increased odds of PTB is supported by additional research [4].

We did not observe any associations between excessive GWG and PTB risk, which is consistent with some literature. For example, a recent systematic review noted that weight gain above the target guidelines was not associated with PTB among underweight women [4]. However, other studies examining the effect of excessive GWG on PTB risk have produced contradictory results. A 7% reduction in odds of preterm labor was seen among women with excessive GWG in the Pregnancy Risk Assessment Monitoring System (PRAMS) [25]. Weight gain above the target guidelines was also associated with reduced odds of PTB among 33,973 underweight and normal weight women in northern China [26]. In contrast, a recent meta-analysis found that obese women with excessive GWG had a 54% increase in medically indicated PTB risk [27], though they did not observe any associations between excessive GWG and spontaneous PTB or total PTB [27]. In our study, we were unable to determine if PTB was spontaneous or medically indicated, which may be one explanation for our null results.

The biologic mechanism linking GWG to PTB is incompletely understood. Maternal weight gain is considered a marker of many physiologic processes, including metabolism and body composition, and may be a marker of nutritional status [6]. Weight gain during pregnancy may also be reflective of macronutrient and micronutrient deficiency, which may be on the casual pathway to PTB [6] and are associated with reduced fetal growth [28]. For example, zinc deficiency is associated with lower appetite and thus low weight gain could be used as a marker for zinc deficiency [6]. Alternatively, low weight gain may be an intermediate for anemia. Anemia is associated with an increased risk of PTB and approximately 50% of women with low weight gain are anemic [6]. Low GWG could also lead to an increase in inflammation and poor expansion of plasma volume, both of which are underlying mechanisms on the causal pathway to PTB [6]. Lastly, low weight gain may be stimulating cortisol production, which would lead to an increase in corticotropin-releasing-hormone (CRH) and prostaglandin and increased susceptibility to uterine contractions [29].

An important strength of this study was that it was conducted using a large set of population-wide data from Puerto Rico. Vital statistics data is routinely collected in a standardized manner and covers nearly all births occurring in Puerto Rico. To date, much of the research on GWG and PTB in Hispanic populations has not differentiated between Hispanic subgroups. Puerto Ricans are a unique and understudied population, as they are a large Hispanic subgroup and are U.S. citizens. Dietary factors are strong predictors of GWG [12] and total fat intake is higher among Puerto Rican populations in the U.S. compared to individuals of Mexican, Dominican, and Central and South American descent [30]. The rates of PTB are also much higher among U.S. based Puerto Ricans relative to their Mexican and Central and South American counterparts [31]. This further emphasizes the need to specifically examine potentially modifiable risk factors leading to adverse birth outcomes in this population. Previous research among women in Puerto Rico has indicated that infants born to women with low and excessive GWG have decreased weight gain between 4 and 6 months compared to infants born to women with adequate weight gain during pregnancy [32]. Excessive GWG has also been associated with increased odds of hypertensive disorders during pregnancy in a predominantly Puerto Rician population [33]. Obese women in Puerto Rico are also more likely to have metabolic syndrome compared to underweight and normal weight women [34].

Our results should be interpreted in light of some limitations. Though birth certificate data is a valuable resource for tracking and analyzing maternal and infant health at the state and national levels, the reliability and validity of it has been of long-standing concern. To the best of our knowledge, there has been no previous assessment addressing the reliability and variation of birth certificate data across different regions of Puerto Rico. Additionally, pre-pregnancy height and weight and weight at delivery were based on what was provided in the birth certificate. Given the nature of birth certificate data, we are unable to determine if this information was based on self-report or abstracted from a medical record. Self-reported measures may be subject to social-desirability bias, as women may report a lower weight [35]. Furthermore, we were not able to assess the rate of gestational weight gain across pregnancy and while we did standardize our GWG measure to a 40-week gestational duration, there may be differences in the rate of weight gain among women who delivered preterm and full term. We were also unable to differentiate between spontaneous and medically indicated PTB and GWG may be contributing to PTB through separate pathways. This is important because previous literature has shown that excessive GWG is more strongly associated with spontaneous, as opposed to medically indicated, PTB [36]. To identify underlying etiologic factors, future studies should focus on understanding the differences between PTB subtypes and various risk factors that could be associated with GWG. Additionally, we do not know if gestational age was based on last menstrual period (LMP) or best obstetrical estimate. Biases may exist between the two and it is suggested that LMP is inaccurate by approximately one week [37]. Our study population also only included births occurring in Puerto Rico and thus our results may not be generalizable to other study populations. Lastly, it has been suggested that these certain GWG cut points are too stringent for underweight women and that these women cannot achieve adequate GWG according to these targets. However, GWG categories in our study was based on current IOM classifications which are the most up to date recommendations made by both medical and public health agencies for pregnant women in the U.S.

## Conclusions

Results from our study indicate that GWG is an important contributor to PTB in Puerto Rico. The associations between low GWG and PTB were strongest for underweight women, although associations persisted within all PP-BMI categories except obese women. In addition to GWG, it is important to consider PP-BMI as an additional modifiable risk factor in relation to adverse pregnancy outcomes. Interventions targeted at identifying women with an unhealthy weight and implementing healthy lifestyle changes prior to conception may help to reduce the rate of PTB in Puerto Rico. Future research in this population should examine other factors that may be contributing to GWG, such as an unhealthy diet, and explore the biologic pathways through which GWG may be contributing to PTB with a focus on differentiating between spontaneous and medically indicated births.

## Data Availability

The data that support the findings from this study are available from the Puerto Rico Department of Health.

## Abbreviations

PTB: Preterm birth
GWG: Gestational weight gain
BMI: Body mass index
PP-BMI: Pre-pregnancy body mass index
ACOG: American College of Obstetricians and Gynecologists
WHO: World Health Organization
IOM: Institute of Medicine
SGA: Small for gestational age
AGA: Average for gestational age
LGA: Large for gestational age
OR: Odds ratio
CI: Confidence interval
PRAMS: Pregnancy risk assessment monitoring system
CRH: Corticotropin-releasing-hormone
LMP: Last menstural period
